# Long‐term survival after immunotherapy for uncontrolled locally advanced temporal bone squamous cell carcinoma followed by chemoradiotherapy: A case report

**DOI:** 10.1002/pro6.1233

**Published:** 2024-06-28

**Authors:** Jin Yan, Jiangdong Sui

**Affiliations:** ^1^ Radiation Oncology Center Chongqing University Cancer Hospital Chongqing China

**Keywords:** immunotherapy, radiotherapy, temporal bone malignancy

## Abstract

Temporal bone squamous cell carcinoma (TBSCC) is a rare and invasive malignant tumor. The common predisposing factors include a history of local radiotherapy and chronic suppurative otitis media. The current treatment approach for TBSCC primarily involves surgery, followed by adjuvant radiotherapy and chemotherapy, based on T staging and high‐risk factors. Although patients with early‐stage TBSCC have a high survival rate after treatment, the majority of patients are diagnosed in the intermediate to advanced stages, with extensive tumor involvement, posing challenges for surgical intervention. Definitive chemoradiotherapy (CRT) serves as a viable alternative for unresectable tumors. Constraints in administering curative radiation doses, due to the tolerance of surrounding organs, can lead to uncontrolled tumor growth. Although programmed cell death 1 inhibitors have demonstrated efficacy in head and neck squamous cell carcinoma and cutaneous squamous cell carcinoma, their application in TBSCC remains underexplored. Herein, we report a case of a 47‐year‐old man diagnosed with unresectable advanced and localized TBSCC. Following inadequate tumor control with primary chemoradiotherapy, immunotherapy was initiated, resulting in disease remission within a follow‐up period of > 4 years.

## Graphical abstract

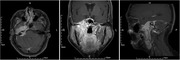

## 1 INTRODUCTION

Temporal bone malignancies (TBMs) are extremely rare tumors, with an incidence rate ranging from one to six cases per million individuals in the general population^1^, ^2^, constituting only 0.2% of all head and neck tumors^3^. Squamous cell carcinoma is the predominant pathological type of temporal bone malignancy, accounting for approximately 80% of all cases. Other less common pathological types include basal cell carcinomas, adenoid cystic carcinomas, neuroendocrine carcinomas, adenocarcinomas, and hematologic tumors^4^, ^5^, ^6^. The major risk factors for temporal bone squamous cell carcinoma (TBSCC) include a history of local radiotherapy, exposure to ultraviolet radiation, and immunosuppression. Other potential associations include recurrent chronic suppurative otitis media and human papillomavirus infection^7^, ^8^. Currently, surgery serves as the mainstay of treatment for temporal bone malignancies, supplemented by radiotherapy and chemotherapy as adjunctive therapeutic modalities. Despite advancements in surgical techniques, radiotherapy, imaging, and other technologies, the 5‐year survival rates of T1–T2 TBSCC and T3–T4 TBSCC are 81.9% and 47.5%^9^, respectively. Nevertheless, owing to the rarity of these tumors and the limited number of case reports and clinical studies, coupled with the complex local anatomy, diagnosing and treating temporal bone malignancies present substantial challenges. Immunotherapy, an emerging treatment modality, is progressively gaining popularity for head and neck squamous cell carcinoma and cutaneous squamous cell carcinoma. Herein, we present a case of locally advanced TBSCC initially deemed unresectable and demonstrated local progression after primary chemoradiotherapy. Subsequently, the patient achieved a progression‐free survival (PFS) of four years after treatment with PD‐1 inhibitors.

## 2 CASE PRESENTATION

**FIGURE 1:**
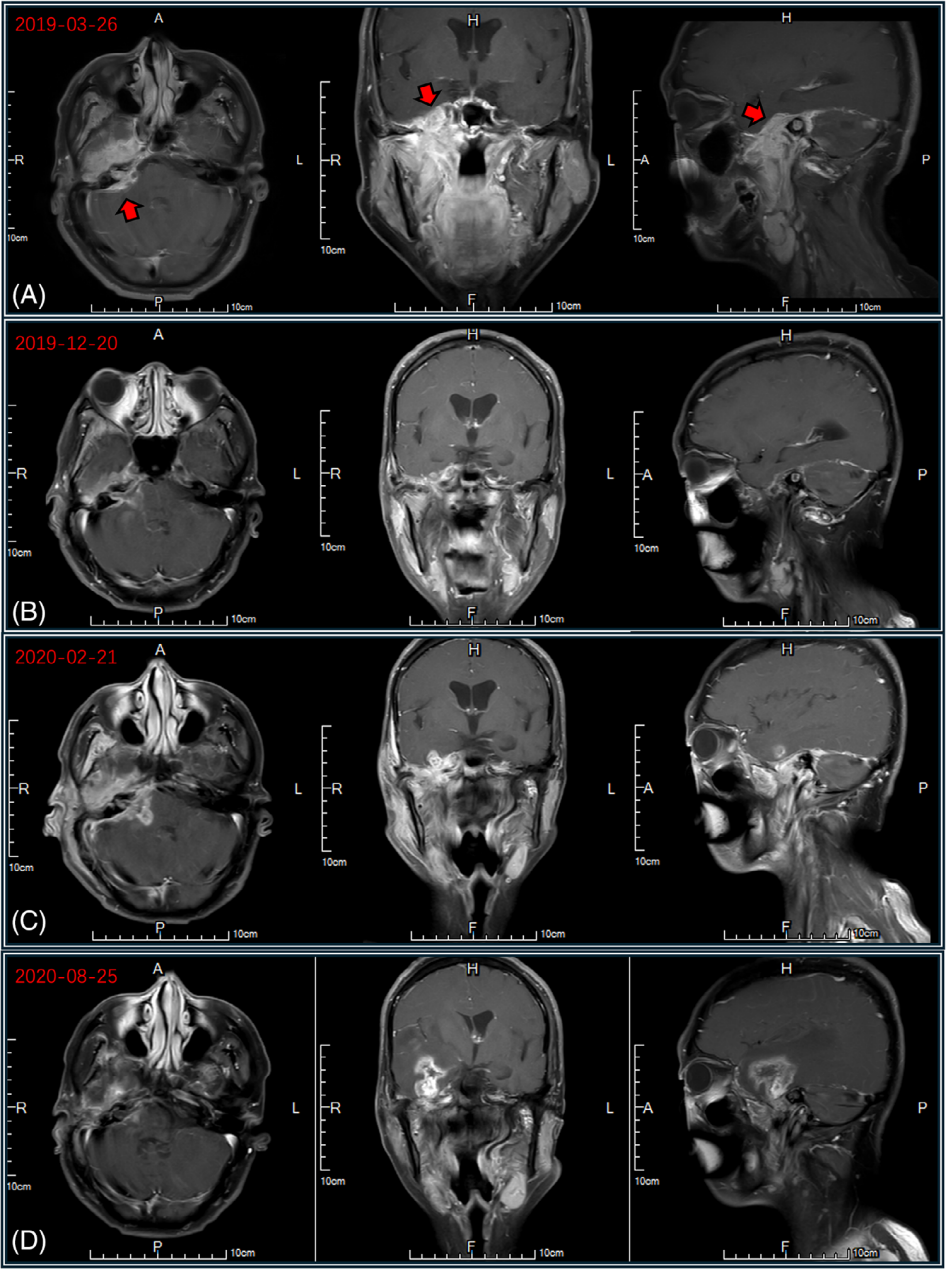
Changes in the tumor observed on magnetic resonance imaging scans at different time points. A) Before treatment. B) After the initial treatment. C) Progression after treatment. D) After carboplatin and 5‐fluorouracil chemotherapy combined with pembrolizumab.

**FIGURE 2:**
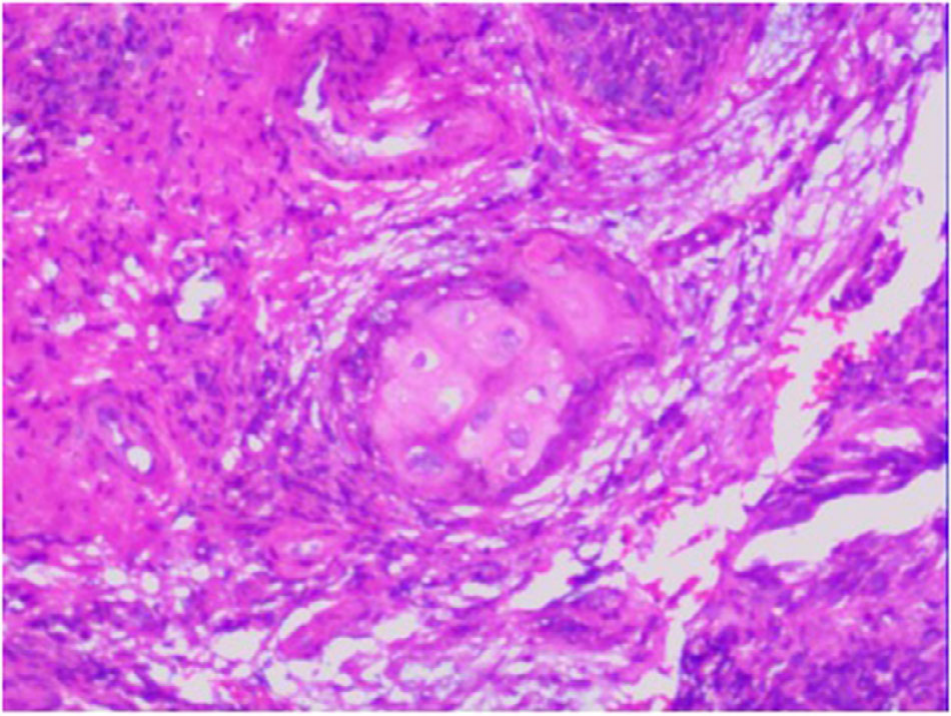
Transnasal endoscopic biopsy of the right nasopharyngeal wall revealed infiltrating squamous cell carcinoma characterized by nest‐like formations of mildly to moderately dysplastic squamous cells. (hematoxylin and eosin, 200×).

In September 2018, a 47‐year‐old man presented with a history of recurrent purulent discharge from the right ear persisting for over 30 years. As the patient was initially suspected with right‐sided purulent otitis media, right mastoidectomy, facial nerve decompression, and atticotomy were performed. Postoperative histopathological examination revealed chronic purulent inflammation in the right middle ear with marked atypical hyperplasia of the squamous epithelium. Although purulent discharge from the right ear slightly decreased postoperatively by March 2019, the patient subsequently reported worsening right ear pain and vertigo. Physical examination revealed right‐sided facial paralysis, indicative of oculomotor, trochlear, abducens, and trigeminal nerve damage (including the disappearance of the right pupil light reflex, medial strabismus, and reduced pain and temperature sensation on the right side of the face). Temporal magnetic resonance imaging (MRI) demonstrated increased soft tissue density in the right ear, parapharyngeal, and pterygopalatine fossa regions, measuring approximately 5 cm × 3.5 cm × 3.4 cm, with the lesion center located in the right temporal bone (Figure 1A). As no definitive tumor cells were found in the ear, a biopsy of the rough mucosa on the right lateral wall of the nasopharynx was performed using nasal endoscopy. The biopsy revealed an infiltrative, moderately differentiated squamous cell carcinoma that tested negative for Epstein–Barr virus (EBV)‐encoded small RNA (Figure 2). The EBV DNA level was < 500 copies/mL. Based on the clinical history and tumor location, a diagnosis of right‐sided TBSCC was established, with no evidence of lymph node or distant organ metastases upon systemic examination.

**FIGURE 3:**
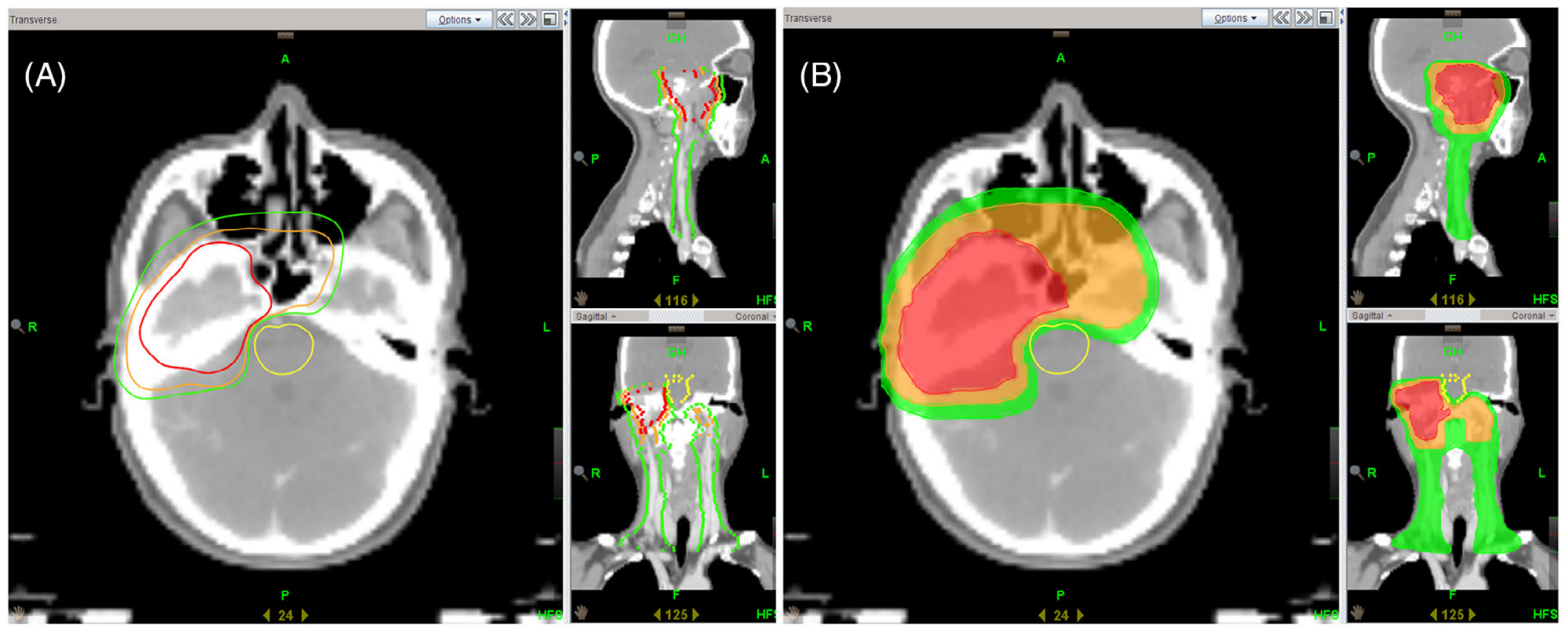
Target areas and plan description. A) Delineation of the GTV (red), CTV_60(orange), and CTV_50(green). B) The distribution of 100% of the PTV_66(red), PTV_60 (orange), and PTV_50 (green) shown in color wash. GTV, gross tumor volume; CTV, clinical target volume; PTV, planning target volume.

In April 2019, the patient underwent external beam radiotherapy after completing two cycles of neoadjuvant chemotherapy with paclitaxel (135 mg/m^2^) and nedaplatin (75 mg/m^2^). The radiotherapy plan was generated using the TomoHD Planning Station (version 5.1.1.6, Accuray Inc.) by employing helical intensity modulation. Pretreatment MRI scans were fused with localization CT scans to delineate the gross tumor volume (GTV). The GTV was expanded by 5−10 mm to form clinical target volume (CTV)_60, encompassing the entire nasopharynx and a submucosal depth of 5−10 mm. CTV_60 was further expanded geometrically by 5 mm in all directions and selectively included bilateral cervical lymph node levels II–V to form CTV_50. The expansion distance of the CTV can be reduced to 1 mm in proximity to the critical organs. Ultimately, the expansions of 3−5 mm for the GTV, CTV_60, and CTV_50 resulted in the generation of planning target volume (PTV)_66, PTV_60, and PTV_50, respectively, delivering a fractionated dose of 2 Gy per session with a gradually shrinking field technique (target areas and plan depicted in Figure 3). The dosimetric details of the organ at risk are provided in Table 1. To mitigate ototoxicity, concurrent chemotherapy with 120 mg of nedaplatin (90 mg/m^2^) was administered during RT. After one cycle of chemotherapy, the patient experienced grade III vomiting, possibly due to the increased chemotherapy dosage and radiotherapy‐induced cerebral edema. Consequently, the patient declined further chemotherapy and received 200 mg/w of nimotuzumab concurrently for four cycles. Following the completion of radiotherapy, the patient underwent four additional cycles of adjuvant chemotherapy with the same paclitaxel and nedaplatin regimens. In December 2019, reassessment revealed partial remission (Figure 1B).

**TABLE 1:** Dose‒volume parameters of the organs at risk.

OAR	Mean dose (Gy)	Maximum dose (Gy)	Others
Brainstem	–	59.80	–
Spinal cord	–	24.35	–
Chiasm	–	53.1	–
Left optic nerve	–	25.27	–
Right optic nerve	–	44.13	–
Left temporal lobe	–	64.36	–
Right temporal lobe	–	68.74	V_65Gy _= 37.84%
Left parotid	24.88	–	D_50% _= 24.8 Gy
Right parotid	42.81	–	D_50% _= 42.73 Gy

Abbreviations: OAR, organ at risk; V_65Gy_, % volume receiving 65 Gy; D_50%_, Gy received at 50% volume.

**FIGURE 4:**
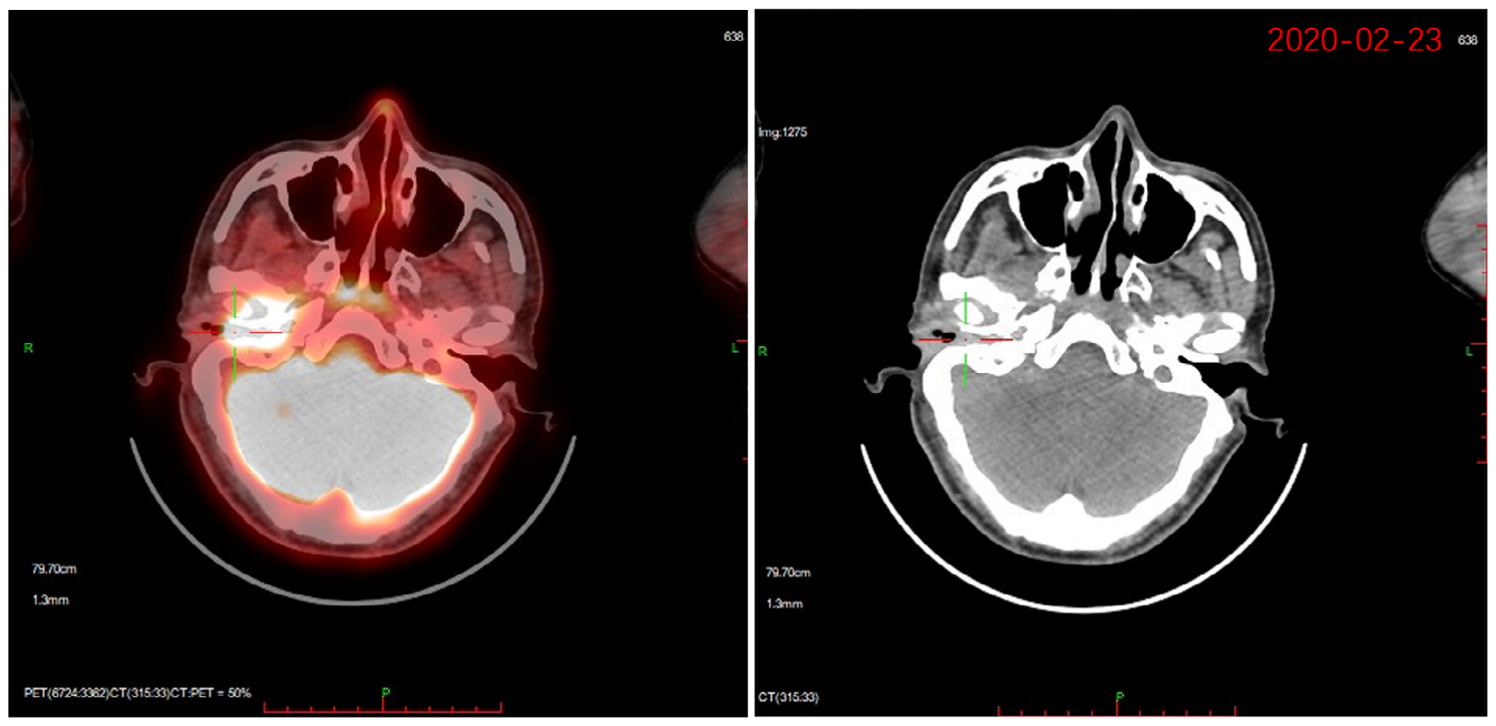
18F‐FDG PET‐CT reveals a slight increase in soft tissue density in the right ear region with elevated metabolism, with an SUVmax of approximately 10.8. ^18^F‐FDG, ^18^F‐fluorodeoxyglucose; PET‐CT, positron emission tomography‐computed tomography.

**FIGURE 5:**
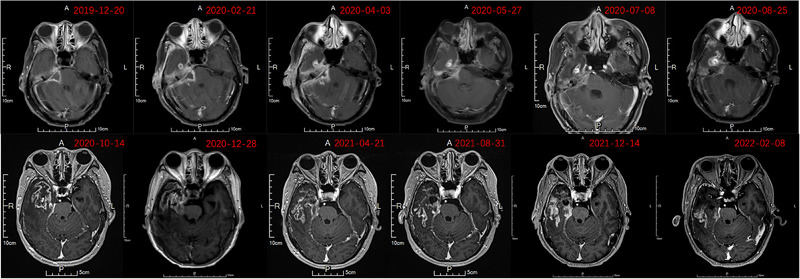
T1‐weighted enhanced MRI revealed significant enhancement in the right cerebellopontine angle and temporal lobe regions, corresponding in size and shape to the irradiation field of the therapy. MRI, magnetic resonance imaging.

**FIGURE 6:**
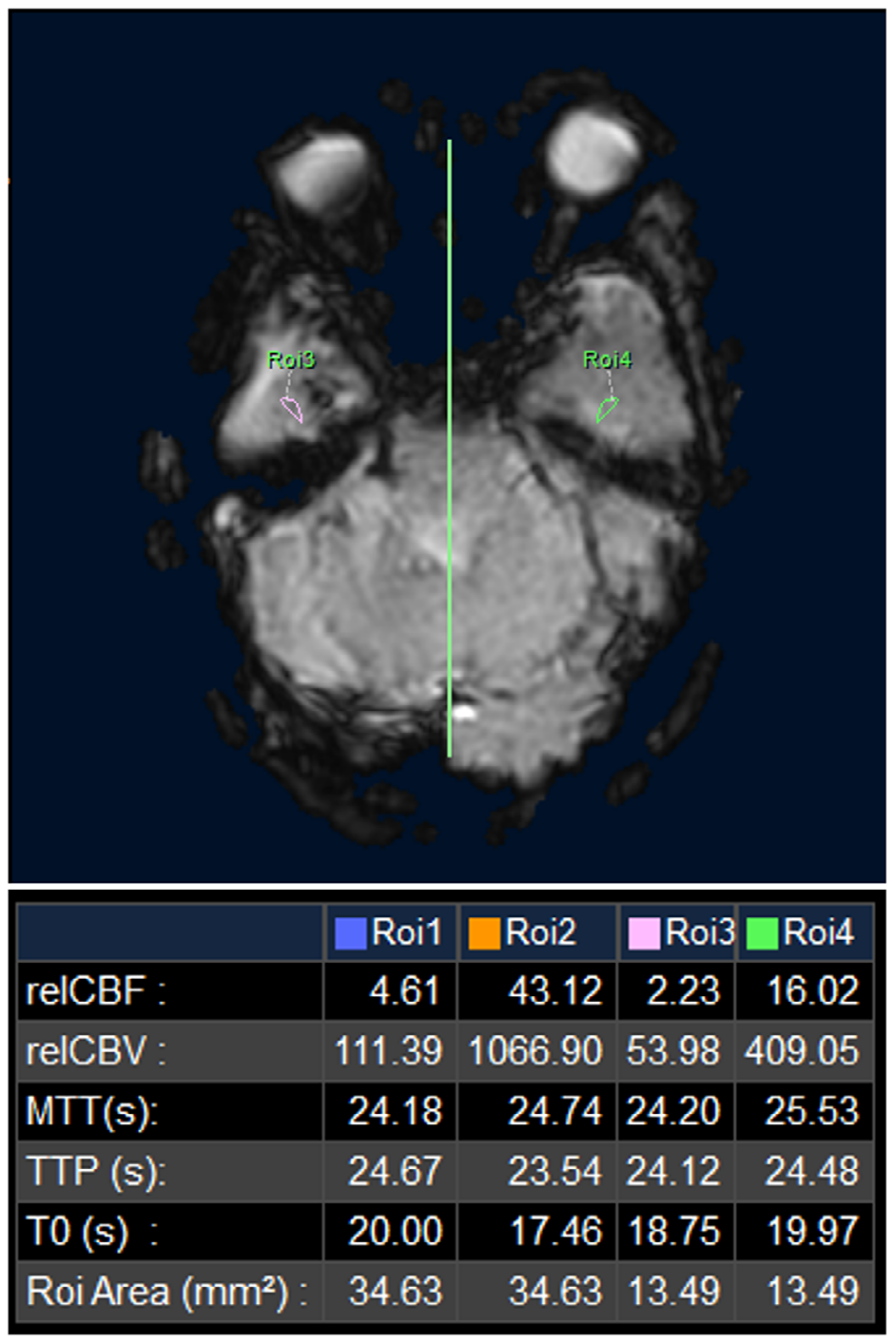
Perfusion‐weighted imaging of the head reveals decreased blood flow perfusion in the area with abnormal signal in the right temporal lobe compared with the mirrored area.

In February 2020, the patient presented with a right‐sided headache accompanied by purulent discharge from the right ear. Positron emission tomography (PET)‐CT revealed a soft tissue shadow in the right ear with an SUV_max_ of 10.8 (Figure 1C and Figure 4). Cytological examination of ear canal secretions confirmed the presence of squamous cell carcinoma cells, indicating local disease progression. Blood specimen analysis revealed a tumor mutation burden of 12.48 Muts/Mb. In March 2020, carboplatin (area under the curve: 5 mg/mL·min) and 5‐fluorouracil (750 mg/m^2^ per day for 5 consecutive days) combined with pembrolizumab (200 mg) were administered every 3 weeks for six cycles. After treatment completion, the headache subsided, ear drainage significantly decreased, and MRI demonstrated tumor regression (Figure 1D). Subsequently, pembrolizumab (200 mg every 21 days) was administered as maintenance therapy. Prior to immunotherapy, cranial MRI revealed significant enhancement changes with edema in the right cerebellopontine angle, extending to the right temporal lobe (Figure 5). Despite the pronounced intracranial lesions, the patient remained asymptomatic. After declining the intracranial lesion biopsy, no further medication was administered. Subsequent cranial MRI scans over two years showed a gradual reduction in enhancement and edema in the affected areas, but without complete resolution. Considering the similarity in shape and distribution between intracranial lesions and high‐dose radiation areas, perfusion‐weighted imaging indicated low perfusion in the enhanced area (Figure 6). Contrary to the progressive enlargement of the intracranial lesions after immunotherapy, symptom relief was observed, leading to a diagnosis of radiation‐induced brain injury. Treatment was discontinued in November 2021 due to the occurrence of grade 3 immune‐related dermatitis, which gradually improved following symptomatic management. Pembrolizumab immunotherapy was resumed in February 2022. In September 2022, after the 10th cycle of pembrolizumab, the patient experienced approximately 100 mL of bleeding from the right external auditory canal, which resolved with compression therapy. Subsequently, immunotherapy was discontinued. As of March 2024, the patient's condition remained stable, with regular follow‐up visits.

## 3 DISCUSSION

TBSCC is a rare malignancy with limited case reports and clinical studies, resulting in the lack of standardized treatment protocols^10^, ^11^. However, surgical intervention remains the cornerstone of TBSCC management^6^. Due to its aggressive nature^12^, early‐stage (T1/T2) temporal bone malignancies are primarily managed with surgical resection, followed by adjuvant therapy based on high‐risk factors. For locally advanced disease (T3/4), a multimodal approach involving surgery followed by adjuvant radiotherapy and chemotherapy is the mainstay of treatment. Emerging evidence suggests that concurrent chemoradiotherapy may achieve outcomes comparable to those of surgery combined with adjuvant radiotherapy^13^, ^14^. In a meta‐analysis conducted by Krystelle et al.^9^, no significant difference was observed in the 5‐year survival rates between patients with T1/T2 TBSCC who underwent radical surgical resection and those who received radiotherapy alone (100% vs. 81.3%, *P *= 0.103). Similarly, for advanced cases (T3/T4), no significant difference was found in the 5‐year survival rates among patients who underwent postoperative chemoradiotherapy, postoperative radiotherapy, and definitive chemoradiotherapy (50% vs. 53.3% vs. 58.1%, respectively, *P *= 0.767−1.000). Therefore, concurrent chemoradiotherapy may be considered an alternative option for patients with locally advanced T3/T4 TBSCC who are unsuitable for surgery or have contraindications to surgery.

With regard to the radiation dosage for TBSCC, postoperative external irradiation typically ranges from 50 to 60 Gy in patients with T2–T4 tumors^15^. In cases with positive surgical margins, the minimum dose is often increased to 66 Gy^16^. Prabhu et al. suggest administering 60−66 Gy to patients with negative surgical margins and 68−72 Gy to those with positive margins^17^. Hashi et al. advocated a dosage of 65−75 Gy for radical radiotherapy alone^18^. The temporal bone is situated within the middle cranial fossa in close proximity to the temporal lobe, cerebellum, and brainstem. The Radiation Therapy Oncology Group recommends a Dmax of ≤60 Gy or a V65 of ≤1% as a tolerance limit for the temporal lobe. Research by Hsiao et al. indicated a more significant decline in cognitive function when the average dose to the temporal lobe exceeded 36 Gy or when the V60 exceeded 10%^19^. In the present case, despite the administration of radiation therapy at a dose of 66 Gy/33 fractions with four cycles of adjuvant chemotherapy for tumor lesions, local control was not achieved. Radiation‐induced damage to the cerebellopontine angle and temporal lobe was evident; however, the patient remained asymptomatic. Therefore, for extensively invasive TBSCC that cannot be surgically excised, localized palliative radiotherapy may be more appropriate for achieving local control, despite the potential need for dose escalation.

In 2016, based on the milestone clinical trials Keynote‐012 and Checkmate‐141^20^, ^21^, the Food and Drug Administration approved the use of pembrolizumab and nivolumab for the treatment of recurrent or metastatic head and neck squamous cell carcinoma (HNSCC), marking a new era in immunotherapy for HNSCC. The Keynote‐048 study further confirmed the efficacy of pembrolizumab in combination with chemotherapy as first‐line treatment for recurrent or metastatic HNSCC^22^. Additionally, pembrolizumab monotherapy was deemed suitable for patients with a combined positive score (CPS) of > 20. To date, no clinical studies have investigated the use of PD‐1 inhibitors for the treatment of TBSCC. However, considering the significant efficacy of PD‐1 inhibitors in several cases of locally advanced or recurrent cutaneous squamous cell carcinoma of the ear and temporal region in recent years^23^, ^24^, ^25^, in this particular case, after confirming local uncontrolled disease, the patient received six cycles of carboplatin and 5‐fluorouracil (PF) chemotherapy combined with pembrolizumab immunotherapy, followed by pembrolizumab maintenance therapy. Despite experiencing grade III immune‐related dermatitis during treatment, which led to temporary treatment discontinuation for four months, the resumption of immunotherapy resulted in long‐term disease control. This may be attributed to the high infiltration of T lymphocytes in the tumor microenvironment, potentially enhancing the immunotherapeutic efficacy of PD‐1 inhibitors in TBSCC that have undergone malignant transformation due to long‐standing chronic otitis media. If TBSCC primarily manifests as a “hot” tumor, it remains unclear whether neoadjuvant immunotherapy can reduce tumor staging, making it easier to achieve negative surgical margins for operable cases. Additionally, whether immunotherapy can prolong PFS and improve the quality of life of patients with inoperable advanced diseases is poorly understood. These important issues warrant further investigation.

## 4 CONCLUSION

Long‐term disease remission of locally advanced TBSCC with uncontrolled focal lesions was achieved using immunotherapy combined with chemotherapy. For patients with inoperable locally advanced TBSCC, further prospective clinical studies are needed to determine whether the use of PD‐1 inhibitors can reduce the radiation dosage and limit the irradiation range.

## CONFLICT OF INTEREST DECLARATION

The authors declare no conflict of interest.

## ETHICS STATEMENT AND INFORMED CONSENT STATEMENT

Written informed consent was obtained from a legally authorized representative for anonymized patient information in this case report to publish this paper. Approval to report this case was obtained from the Ethics Committee of Chongqing University Cancer Hospital (Approval Number: CZLS2024151‐A).
